# Editorial: Integrating nutrition in cancer therapy: approaches to improve patient outcomes and survival

**DOI:** 10.3389/fonc.2026.1881486

**Published:** 2026-06-10

**Authors:** Aurora Mirabile, Nerina Denaro, Vanesa Gregorc

**Affiliations:** 1 Department of Otolaryngology, Istituto di Ricovero e Cura a Carattere Scientifico (IRCCS) Ospedale San Raffaele, Milan, Italy; 2 Oncology Unit, Fondazione Istituto di Ricovero e Cura a Carattere Scientifico (IRCCS) Ca’ Granda Ospedale Maggiore Policlinico, Milan, Italy; 3 Unit of Oncology and Haematology, Candiolo Cancer Institute, Fondazione del Piemonte per l’Oncologia (FPO)-Istituto di Ricovero e Cura a Carattere Scientifico (IRCCS), Candiolo, Italy

**Keywords:** cancer metabolism, cancer survivorship, immunonutrition, malnutrition, nutrition in oncology, nutritional support, precision oncology, sarcopenia

The integration of nutrition into cancer care has emerged as a crucial component in optimizing patient outcomes, yet it remains inconsistently implemented across clinical settings. This Research Topic, *Integrating Nutrition in Cancer Therapy: Approaches to Improve Patient Outcomes and Survival*, brings together multidisciplinary contributions highlighting the role of nutritional strategies across the cancer continuum, with particular attention to the interplay between metabolism, immune function, and treatment response.

Malnutrition and metabolic dysregulation are highly prevalent among oncology patients, particularly in those with head and neck and gastrointestinal malignancies, where symptoms such as dysphagia xerostomia and other acute treatment-related toxicities significantly impair oral intake. Even early weight loss exceeding 5% has been associated with poorer progression-free survival, underscoring the prognostic relevance of nutritional status. Beyond its impact on prognosis, malnutrition contributes to increased morbidity, treatment delays, and unplanned hospitalizations (1).

The contributions collected in this Research Topic reinforce that nutrition should not be regarded merely as supportive care but rather as a fundamental component of cancer therapy. This paradigm shift is highlighted in *“Embedding nutrition into routine oncology care: about time?”*, while epidemiological evidence linking high body mass index to cancer burden further underscores the broader relevance of nutritional factors across the disease spectrum.

A central theme emerging from recent contributions is the critical importance of early nutritional screening and risk stratification in oncology. Predictive models—such as nomograms for malnutrition in colorectal cancer and composite indices integrating inflammatory and dietary parameters in gastric cancer—demonstrate how combining nutritional and biological variables can enhance prognostic accuracy. In parallel, data-driven approaches are increasingly being used to identify emerging research trends and optimize personalized nutritional strategies in cancer care.

Recently, the American Society for Parenteral and Enteral Nutrition published updated recommendations emphasizing the need for proactive and structured nutritional management. These include early initiation of enteral nutrition when oral intake is inadequate, commencement of nutritional support within 24 hours after surgery, weekly dietitian consultations during radiotherapy, systematic malnutrition screening using validated tools, interdisciplinary models of care, and dietitian involvement both before and after surgery. Additionally, specific targets are proposed for protein intake (1.2–1.5 g/kg/day) and energy intake (≥30 kcal/kg/day) (2).

Despite these advances, the awareness and implementation of such recommendations remain insufficient in clinical practice. Treatment-related toxicities frequently exacerbate nutritional impairment, highlighting the need to consider nutrition as a core component of oncologic care rather than a supportive measure. Accordingly, nutritional intervention should be prescribed as an early therapeutic strategy, ideally preceding surgery or chemoradiotherapy.

In this context, Casalone et al. highlighted the potential role of immunonutrition in the preoperative setting. However, more recent evidence challenges its added value. A phase III trial reported no significant differences between standard perioperative enteral nutrition and immunonutrition in terms of anthropometric parameters, biochemical markers, length of hospital stay, or complication rates (3). These findings suggest that the presumed benefits of specific nutrients—such as arginine, glutamine, omega-3 fatty acids (EPA/DHA), and nucleotides—in enhancing immune response, reducing inflammation, and improving wound healing may not translate into consistent clinical advantages.

Rather, emerging evidence indicates that the quality, timing, and duration of nutritional support may be more relevant determinants of outcome than the specific composition of immunonutrient-enriched formulas (4). Supporting this perspective, Belak et al., in a large retrospective cohort study of over 900 patients, demonstrated that prolonged medical nutritional support (>6 months) was associated with improved outcomes (5).

The interplay between metabolism, inflammation, and tumor biology represents another key focus. Cancer-related metabolic alterations contribute to muscle wasting and functional decline, with direct implications for treatment outcomes. The study on body composition and acute kidney injury in head and neck cancer illustrates how nutritional parameters can predict treatment-related toxicity (Trevisani et al.) (6). Additional biomarkers—including branched-chain amino acids, systemic inflammatory indices, and hormonal proxies such as the fT3/fT4 ratio—are further expanding the understanding of cancer-associated metabolic alterations. These parameters provide insight into the complex interplay between tumor biology, host metabolism, and systemic inflammation. In parallel, morpho-functional assessments (e.g., body composition analysis, muscle strength, and physical performance) have demonstrated prognostic relevance in complex clinical scenarios such as hematopoietic stem cell transplantation, where metabolic reserve and functional status significantly influence outcomes.

These interactions acquire particular importance in the era of immunotherapy, as nutritional and metabolic status may modulate response to immune checkpoint inhibitors, potentially affecting both treatment efficacy and toxicity profiles.

Nutritional interventions—ranging from enteral nutrition and oral nutritional supplements to targeted dietary modulation—are widely investigated across oncologic settings. A recent systematic review and meta-analysis demonstrated that high-protein oral supplementation is associated with a reduction in postoperative complications and length of hospital stay. In surgical oncology, the integration of enhanced recovery protocols with early enteral nutrition has shown clinically meaningful benefits, particularly in gynecologic malignancies.

Moreover, real-world evidence underscores the clinical relevance of sustained nutritional care. Improved survival outcomes have been reported in patients with head and neck cancer receiving long-term nutritional support alongside systemic therapies, reinforcing the concept that continuous and structured nutritional intervention represents a key determinant of oncologic outcomes.

Innovative approaches, including ketogenic and fasting-mimicking diets and long-term nutraceutical strategies, highlight the potential of metabolic modulation. At the same time, behavioral and social dimensions remain essential: caregiver involvement, self-efficacy–based interventions, and structured lifestyle programs in cancer survivors all contribute to improved adherence and outcomes.

Collectively, these contributions reflect a paradigm shift toward recognizing nutrition as a cornerstone of precision oncology, spanning prevention, treatment, and survivorship. However, important challenges remain, including variability in clinical practice, limited access to specialized nutritional care, and the need for high-quality evidence from well-designed clinical trials.

Future research should refine patient stratification, integrate metabolic and inflammatory biomarkers into clinical decision-making, and evaluate interventions based on clinically meaningful outcomes. Equally important is the translation of existing evidence into standardized care pathways that can be implemented across diverse healthcare systems.

Ultimately, the integration of nutrition into oncology care should no longer be considered optional but rather a core standard of care, one that is as essential as any oncological treatment in improving patient outcomes and survival.
